# Reported outcomes for patients following postoperative periprosthetic femoral fractures: a systematic review

**DOI:** 10.1302/2046-3758.1510.BJR-2025-0734

**Published:** 2026-10-15

**Authors:** Wenjuan Cong, Emily Howell, Tom Krause, Rebecca Fox, Wendy Bertram, Josh Lamb, Hung-Yuan Cheng, Michael Whitehouse, Jonathan Evans, Vikki Wylde, Alex Aquilina

**Affiliations:** 1 Musculoskeletal Research Unit, Bristol Medical School (THS), University of Bristol, Bristol, UK; 2 Exeter Hip Unit, Princess Elizabeth Orthopaedic Centre, Royal Devon University Healthcare NHS Foundation Trust, Exeter, UK; 3 University Hospitals Bristol and Weston NHS Foundation Trust, Bristol, UK; 4 Therapy Department, Royal Devon University Healthcare NHS Foundation Trust, Exeter, UK; 5 National Institute for Health and Care Research Exeter Biomedical Research Centre, University of Exeter Medical School, St Luke’s Campus, Exeter, UK; 6 National Institute for Health and Care Research Bristol Biomedical Research Centre, University Hospitals Bristol and Weston NHS Foundation Trust and University of Bristol, Bristol, UK

**Keywords:** Periprosthetic femoral fracture, Outcomes, Core outcome set, Patient-reported outcome measures, Systematic review, periprosthetic femoral fractures, Hip, bone union, Knee, patient-reported outcome measures (PROMs), clinical trials, arthroplasty surgery, clinician, surgical complications, Short-Form Health Survey

## Abstract

**Aims:**

Postoperative periprosthetic femoral fracture (POPFF) is an increasingly common and morbid complication of arthroplasty. This review aims to determine what outcomes are reported for POPFF and how they are measured, to inform the development of a core outcome set.

**Methods:**

Systematic searches of major databases and clinical trial registries (June 2014 to May 2024) identified studies reporting outcomes for more than five POPFF patients in English or Chinese. Multiple reviewers performed study screening and data extraction. Outcomes were extracted verbatim, collapsed into outcome domain subcategories, and categorized. Outcomes were synthesized descriptively, with subgroup exploration by implant (hip/knee) and economic setting.

**Results:**

A total of 564 studies, comprising 119,445 POPFF patients (mean age 75.0 years (38.5 to 90.0); median percentage of female patients 61.8%), were included. Across the studies, 2,711 individual reported outcomes were collapsed into 671 unique terms within 59 outcome domain subcategories. Outcome reporting was dominated by the physiological/clinical core area (38.7%), followed by life impact (29.3%), resource use (12.8%), adverse events (11.7%), and death (7.6%). The most frequently reported subcategories were bone union/healing, surgical complications, component/prosthesis outcomes, hip function, and mortality/survival. Only 46.4% of outcomes were accompanied by a clear definition or named outcome measurement instrument; for bone union, just 7.4% provided a clear definition, 44.9% were vague, and 47.7% provided none. Health-related quality of life was measured in 6.9% of studies (39 instances) using seven instruments, most commonly the 12/36-Item Short-Form Health Survey questionnaire and EuroQol five-dimension questionnaire. Patterns varied between hip and knee POPFF and between high-income and low- to middle-income settings; one-year mortality was the most frequently reported survival timepoint.

**Conclusion:**

Outcome reporting after POPFF is heterogeneous, clinician-centred, and frequently undefined, with sparse use of patient-reported outcome measures and health-related quality of life instruments. These findings support the development of a POPFF core outcome set and recommended outcome measurement instruments to standardize reporting and promote patient-centred research.

Cite this article: *Bone Joint Res* 2026;15(10):1236–1247.

## Article focus

- What outcomes are reported for postoperative periprosthetic femoral fracture, and how are they defined and measured?
- How do outcome choices vary by implant (hip vs knee) and by economic setting?
- What gaps and inconsistencies need to be addressed to enable robust patient-centred evidence synthesis?

## Key messages

- Outcome reporting is dominated by physiological/clinical endpoints (e.g. union, implant-related events); life impact outcomes are poorly represented in the literature.
- Definitions of outcome measurement instruments (OMIs) are frequently absent or vague.
- The findings support the development of a postoperative periprosthetic femoral fracture-specific core outcome set with a recommended set of feasible OMIs.

## Strengths and limitations

- This is a large, inclusive review across 564 studies from the English and Chinese literature, mapped to the Core Outcome Measures in Effectiveness Trials (COMET) Taxonomy.
- Descriptive synthesis enables the comprehensive cataloguing of outcomes and OMIs.
- In order to maximize outcome capture (data saturation), study methodological quality was not appraised, consistent with reviews focused on outcome mapping rather than effect estimation.

## Introduction

Postoperative periprosthetic femoral fracture (POPFF) is a devastating complication affecting a growing proportion of patients following hip and knee arthroplasty.^1,2^ POPFF is now recognized as the most common cause for major reoperation following total hip arthroplasty.^3,4^ POPFF carries substantial patient morbidity, with mortality comparable to that of native hip fracture in the long and short term, respectively.^5^ POPFF places marked and increasing pressure on orthopaedic services, particularly in the context of an ageing population and growing arthroplasty volume.^3,4,6^ Treatment is complex, costly, and influenced by fracture morphology, implant stability, and patient physiology. Optimal, evidence-based treatment pathways for these challenging injuries remain unclear.^5,7^

Variation in surgical approach and clinical decision-making is common. National audit data highlight that some centres exclusively transfer patients to specialist units, while others manage POPFFs locally without access to dedicated revision teams or implants.^2^ This variation is mirrored in the literature, with strategies differing between institutions and within centres themselves.^5,7,8^

Scoping reviews of the literature demonstrate considerable heterogeneity in outcome reporting. Most studies report outcomes indicative of surgical success, such as implant survivorship, bone union, or reoperation rates; few include validated patient-reported outcome measures (PROMs) or functional assessments. A lack of standardized outcomes and definitions and inconsistent use of outcome measurement instruments (OMIs) impair evidence synthesis, perpetuate the ongoing risk of outcome reporting bias in the literature, and prioritize surgeon-preferred over patient-centred outcomes.

The development of a core outcome set (COS) will help address these shortcomings. A COS is an agreed-upon standardized set of outcomes that should be measured and reported, at a minimum, in all clinical trials and observational studies in a defined area of health or health care.^9^

A COS for POPFF will provide a framework to standardize research reporting, enabling meta-analysis and benchmarking across studies. This review aims to identify what outcomes are currently reported in the POPFF literature and how these are measured to inform the development of a COS for use in future research and clinical audit.

## Methods

This systematic review was registered on the Prospective Register of Systematic Reviews (PROSPERO CRD42024545138) and reported with the aid of the PRISMA statement (Supplementary Material 1).

### Searches and selection criteria

Searches were conducted across the following databases and trial registries: Web of Science, Embase, PubMed, Physiotherapy Evidence Database (PEDro), Allied and Complementary Medicine Database (AMED), the Cochrane Library, two Chinese academic databases (China National Knowledge Infrastructure and Chinese Science and Technology Periodicals Database (VIP)), ClinicalTrials.gov, ISRCTN – The UK’s Clinical Study Registry, the International Clinical Trials Registry Platform, and the Chinese Clinical Trial Registry, covering the period from June 2014 to May 2024. The detailed search strategy is provided in Supplementary Material 2.

Randomized trials, retrospective and prospective observational studies, qualitative studies, systematic reviews, and trial protocols were included. Studies were restricted to those reporting outcomes for at least five POPFF patients. Publications were limited to the English and Chinese languages, without restrictions on the type of surgery or implant. Patients who had undergone hip or knee arthroplasty surgery or fracture fixation of the femur, and later sustained a POPFF, were included. Intraoperative periprosthetic fractures, native fractures, case reports, biomechanical and modelling studies, studies focusing solely on outcomes of primary arthroplasty or the incidence and risk factors of POPFF, case series or mixed cohorts with fewer than five POPFF patients, and ineligible publication types (e.g. literature reviews that were not systematic reviews, commentaries, editorials, letters, and conference abstracts) were excluded. There were no restrictions on populations or healthcare settings in which studies were conducted. High-income countries (HICs) and low- and middle-income countries (LMICs) were defined according to the Development Assistance Committee list of official development assistance recipients from the Organisation for Economic Co-operation and Development.^10^

### Screening and data extraction

Title and abstract screening was conducted by WC, VW, EH, and TK using EndNote (v. 2025; Clarivate, USA). Abstracts and full texts of potentially relevant references were then reviewed in duplicate by WC, VW, JE, JL, AA, WB, RF, HYC, EH, and TK, to determine eligibility for data extraction based on inclusion and exclusion criteria. Any disagreements were resolved by consensus.

A custom data extraction sheet was developed and piloted using an Excel spreadsheet (Microsoft, USA). Table I details the data fields extracted during full-text review. Outcomes were extracted verbatim, including the outcome definition or OMI and the timepoint of measurement, from the methods or results sections of the included studies. Outcomes were defined where an outcome definition was provided or referenced with a citation. Where studies reported PROMs or physical performance measures without stating what outcomes they intended to measure, an overarching outcome was assigned for the purposes of categorization.

**Table I.** Data fields extracted at full-text review.

Study characteristics	Study outcomes
Study authors	Verbatim wording of outcome
Year of publication	Outcome definition or named outcome measurement instrument used to measure outcome
Study title	Timepoints measured post-intervention
Journal	
Journal reference (volume, issue, and page numbers)	
Study registry number if available	
Study web address	
Country (location of lead institution)	
Economic setting (e.g. high-income country or low- and middle-income country)	
Study design (interventional, observational, systematic review)	
Participant demographic details (number of participants with postoperative periprosthetic femoral fracture, mean age, sex distribution, implant type: hip or knee arthroplasty, mixed or other fixation device, operation: revision, fixation, or mixed)	

Data extraction was completed by WC, VW, AA, RF, WB, EH, TK, and JE, with 20% of the data randomly checked by WC. A formal quality assessment of the included studies was not performed, as the primary aim was to compile all outcomes reported for POPFF patients, regardless of study quality.

### Data synthesis

Characteristics of included studies were summarized. The outcomes were categorized using the Core Outcome Measure in Effectiveness Trials (COMET) Taxonomy into five core areas and 38 outcome domains.^11^ Core areas included mortality/survival, physiological/clinical, life impact, resource use, and adverse events/effects. The COMET Taxonomy of Outcomes is a categorization system designed to provide sufficient granularity and scope for classifying all medical outcomes in COS development.^11^ Within each outcome domain, individual outcomes were grouped and categorized under an outcome domain subcategory, using an iterative approach. The outcome domain subcategory was often representative of the most common outcome wording reported in the literature. The frequency of outcome reporting by outcome domain subcategory was calculated and stratified by implant type, economic setting, and study country.

The frequency of OMI reporting was calculated for POPFFs at a hip or knee arthroplasty, with further stratification by economic setting. The frequency of mortality/survival reporting was stratified by commonly reported postoperative follow-up timepoints.

## Results

A total of 8,592 records were identified; after deduplication (n = 3,811), 4,781 titles/abstracts were screened. Of these, 3,194 were excluded. Full-text assessment excluded 1,023 studies (977 ineligible, 33 inaccessible, and 13 not in English or Chinese), resulting in 564 included studies (Figure 1).

**Fig. 1:**
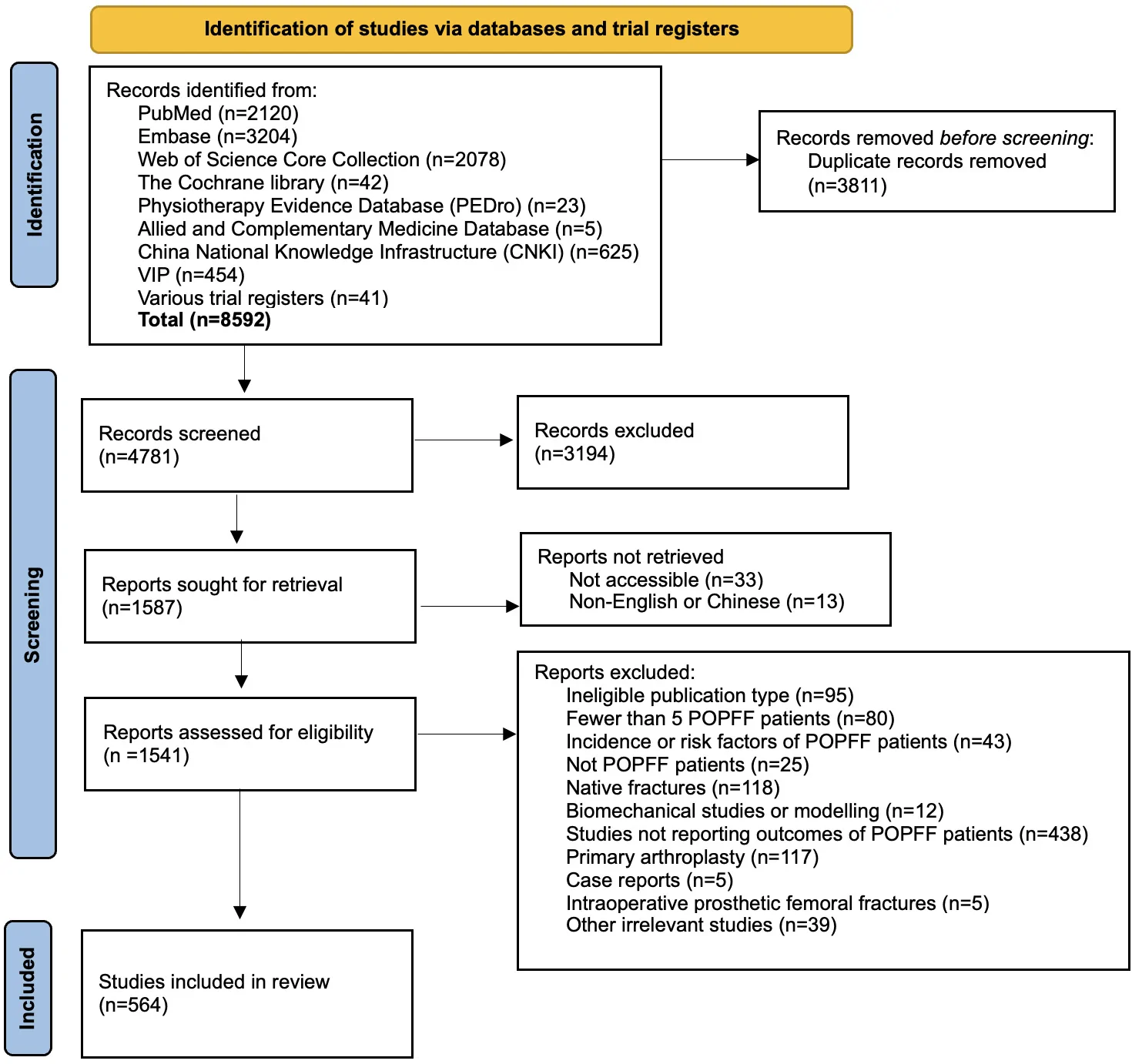
PRISMA chart. POPFF, periprosthetic femoral fracture.

Across 564 studies, 119,445 patients with POPFF were included, with a median sample size of 39 (IQR 19 to 81); the median percentage of patients who were female was 65.5% (IQR 53.9% to 77.7%) and the mean age was 75.0 years (38.5 to 90.0) (Table II). Most records (n = 556) were identified through database searches, with the rest identified from clinical trial registries. The evidence base was predominantly observational (n = 510), with 39 systematic reviews. POPFF associated with a hip arthroplasty was reported in 370 studies (58,755 patients) and knee arthroplasty in 134 studies (20,937 patients). Reported interventions were revision plus fixation in 84,982 patients, revision alone in 20,816, and fixation alone in 13,429.

**Table II.** Summary of studies included in data synthesis.

Variable	Number of studies (% of total studies)	Number of patients
**Article type**		
Database	556 (98.6)	119,164
Trial registry	8 (1.4)	281
**Study design**		
Systematic review	39 (6.9)	31,326
Interventional	15 (2.7)	847
Observational	510 (90.4)	87,272
**Type of implantation**		
Hip arthroplasty	370 (65.6)	58,755
Knee arthroplasty	134 (23.8)	20,937
Others, e.g. mixed (hip and knee), intramedullary nail/sliding hip screw	60 (10.6)	39,753
**Type of operation**		
Revision	108 (19.1)	20,816
Fixation	200 (35.5)	13,429
Mixed	250 (44.3)	84,982
Non-surgical	6 (1.1)	218
**Region**		
East Asia and Pacific	211 (37.4)	8,500
Europe and Central Asia	203 (36.0)	45,049
North America	95 (16.8)	35,919
South Asia	8 (1.4)	253
Latin America and the Caribbean	7 (1.2)	335
Middle East and North Africa	3 (0.5)	54
Multiple regions	37 (6.6)	29,335
**Economic settings**		
High-income countries (n = 30)	348 (61.7)	83,711
Low- and middle-income countries (n = 7)	179 (31.7)	6,399
Mixed	37 (6.6)	29,335
**Country (top 6)**		
China	161 (28.5)	5,806
USA	79 (14.0)	33,852
UK	48 (8.5)	25,648
Italy	31 (5.5)	1,552
South Korea	29 (5.1)	865
Spain	21 (3.7)	2,175

The majority of included studies were observational in design (510; 90.4%), consisting predominantly of retrospective case series, with a smaller number of systematic reviews (39; 6.9%) and interventional studies (15; 2.7%).

Geographically, most patients were from Europe and Central Asia (45,049), North America (35,919), and East Asia and the Pacific (8,500). The largest national cohorts were from the USA (33,852) and the UK (25,648), followed by China (5,806), Spain (2,175), Italy (1,522), and South Korea (865). Where economic setting was reported, most patients were from HICs (83,711) rather than LMICs (6,399).

### Outcomes reported

In total, 2,711 individual outcomes were extracted verbatim, from which 671 unique outcome terms were identified. The 671 outcome terms were grouped under 59 outcome domain subcategories, representing outcomes with the same meaning but different wording. Outcome domain subcategories were then categorized under 17 outcome domains within the four core areas of the COMET Taxonomy of Outcomes. Table III illustrates the number and frequency of outcomes reported in the literature categorized by the outcome domains within the COMET Taxonomy of Outcomes.

**Table III.** Outcome domains identified, and how they were reported in 564 studies on postoperative periprosthetic femoral fracture. Values are presented as n (%).

Outcome domain*	Individual outcomes reported	Unique outcome terms reported	Outcome domain subcategories created	OMIs		
				Definitions stated	Individual patient/clinician-reported outcome measures or PPMs reported	Unique patient/clinician-reported outcome measures or PPMs reported
**Death**						
Mortality/survival (1) (%)	206 (7.6)	26 (3.9)	2 (3.4)	10 (2.6)	0 (0)	0 (0)
**Physiological/clinical**						
Blood and lymphatic system outcomes (2)	87 (3.2)	37 (5.5)	3 (5.1)	15 (3.9)	0 (0)	0 (0)
General outcomes (9)	8 (0.3)	3 (0.4)	3 (5.1)	0 (0)	7 (1.5)	4 (7.4)
Metabolism and nutrition outcomes (14)	1 (0.04)	1 (0.1)	1 (1.7)	0 (0)	0 (0)	0 (0)
Musculoskeletal and connective tissue outcomes (15)	937 (34.6)	341 (50.8)	15 (25.4)	142 (36.7)	49 (10.4)	2 (3.7)
Renal and urinary outcomes (19)	4 (0.1)	1 (0.1)	1 (1.7)	0 (0)	0 (0)	0 (0)
Respiratory, thoracic, and mediastinal outcomes (22)	6 (0.2)	2 (0.3)	1 (1.7)	0 (0)	0 (0)	0 (0)
Skin and subcutaneous tissue outcomes (23)	5 (0.2)	4 (0.6)	2 (3.4)	0 (0)	0 (0)	0 (0)
Total for physiological/clinical (%)	1048 (38.7)	389 (58.0)	26 (44.1)	157 (40.6)	56 (11.9)	6 (11.1)
**Life impact**						
Physical functioning (25)	630 (23.2)	99 (14.8)	10 (16.9)	50 (12.9)	374 (79.4)	39 (72.2)
Emotional functioning/wellbeing (28)	2 (0.07)	2 (0.3)	2 (3.4)	0 (0)	2 (0.4)	2 (3.7)
Global quality of life (30)	47 (1.7)	8 (1.2)	1 (1.7)	0 (0)	39 (8.3)	7 (13.0)
Delivery of care (32)	87 (3.2)	8 (1.2)	2 (3.4)	1 (0.3)	0 (0)	0 (0)
Personal circumstances (33)	27 (1.0)	10 (1.5)	1 (1.7)	12 (3.1)	0 (0)	0 (0)
Total for life Impact, (%)	793 (29.3)	127 (18.9)	16 (27.1)	63 (16.3)	415 (88.1)	48 (88.9)
**Resource use**						
Economic (34)	7 (0.26)	4 (0.6)	2 (3.4)	4 (1.0)	0 (0)	0 (0)
Hospital (35)	174 (6.4)	20 (3.0)	7 (11.9)	8 (2.1)	0 (0)	0 (0)
Need for intervention (36)	166 (6.1)	45 (6.7)	1 (1.7)	13 (3.4)	0 (0)	0 (0)
Total for resource use (%)	347 (12.8)	69 (10.3)	10 (16.9)	25 (6.5)	0 (0)	0 (0)
**Adverse events**						
Adverse events/effects (38)	317 (11.7)	51 (7.6)	5 (8.5)	132 (34.1)	0 (0)	0 (0)
**Total**	2711	671	59	387	471	54

Reported outcomes are shown as the number of all outcomes reported, outcomes reported with unique terminology, and the number of outcome domain subcategories created within each outcome domain. Outcome measurement instruments are presented as the total number of definitions of measurement, named outcome measurement instruments, and unique named outcome measurement instruments.

* Organized by the COMET Taxonomy of Outcomes, core areas, and outcome domains.

COMET, Core Outcome Measures in Effectiveness Trials; OMIs, outcome measurement instruments; PPM, physical performance measures.

Outcomes within the physiological/clinical core area were the most frequently reported (1,048; 38.7%), followed by life impact (793; 29.3%), adverse events (317; 11.7%), resource use (347; 12.8%), and death (206; 7.6%) (Figure 2). The musculoskeletal and connective tissue outcome domain was the most represented (937; 34.6% of all outcomes reported) followed by physical functioning (630; 23.2% of all outcomes reported). Of the 59 outcome domain subcategories, bone union was most commonly reported (392; 14.5%), followed by surgical complications (288; 10.6%), component/prosthesis outcomes (235; 8.7%), hip function (213; 7.9%), and mortality/survival (194; 7.2%). See Supplementary Material 3 for detailed outcome reporting by outcome domain subcategory, categorized using the COMET Taxonomy. Figure 3 shows the most reported outcomes by fracture associated with a hip or knee arthroplasty only, demonstrating that bone union remains the most frequently reported outcome domain subcategory regardless of fracture location/association.

**Fig. 2:**
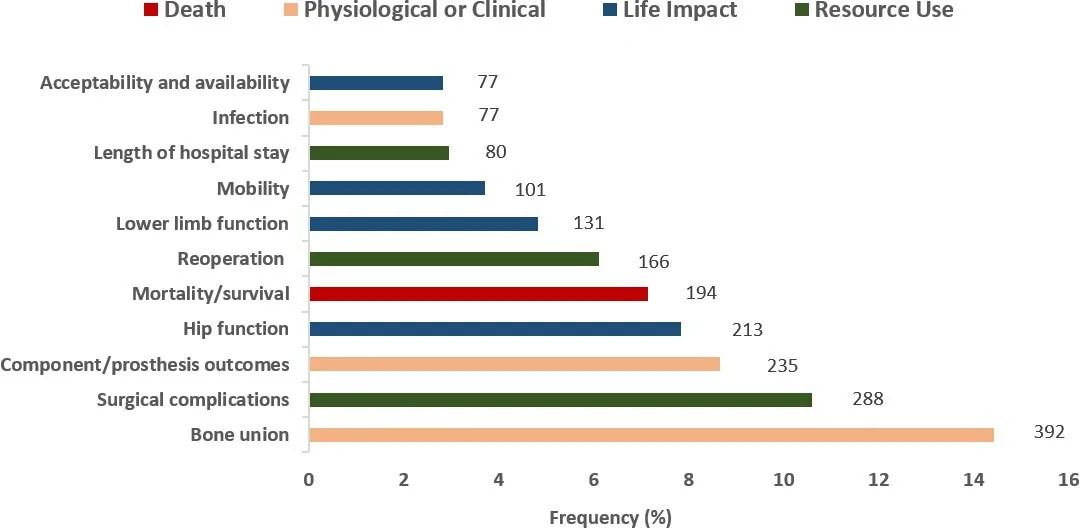
Frequently reported outcomes by core area.

**Fig. 3:**
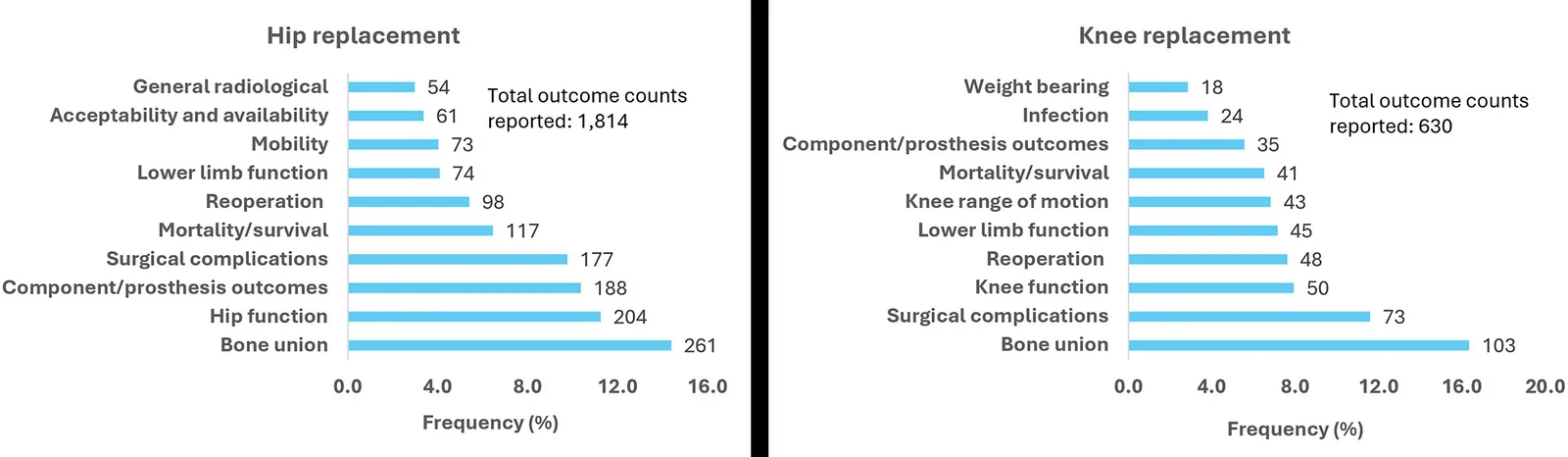
Frequently reported outcomes for postoperative periprosthetic femoral fracture patients following hip and knee arthroplasty.

There is some variation in the frequently reported outcome domain subcategories between HICs and LMICs (Figure 4). In HICs, the most commonly reported were surgical complications (182; 12.2%), mortality/survival (163; 10.9%), and bone union (150; 10.0%), whereas in LMICs they were bone union (221; 12.1%), hip function (129; 10.1%), and component/prosthesis outcomes (107; 7.9%). LMICs also frequently reported pain and discomfort, infection, duration of surgery, and dislocation, while HICs more often reported reoperation, length of hospital stay, and radiological outcomes.

**Fig. 4:**
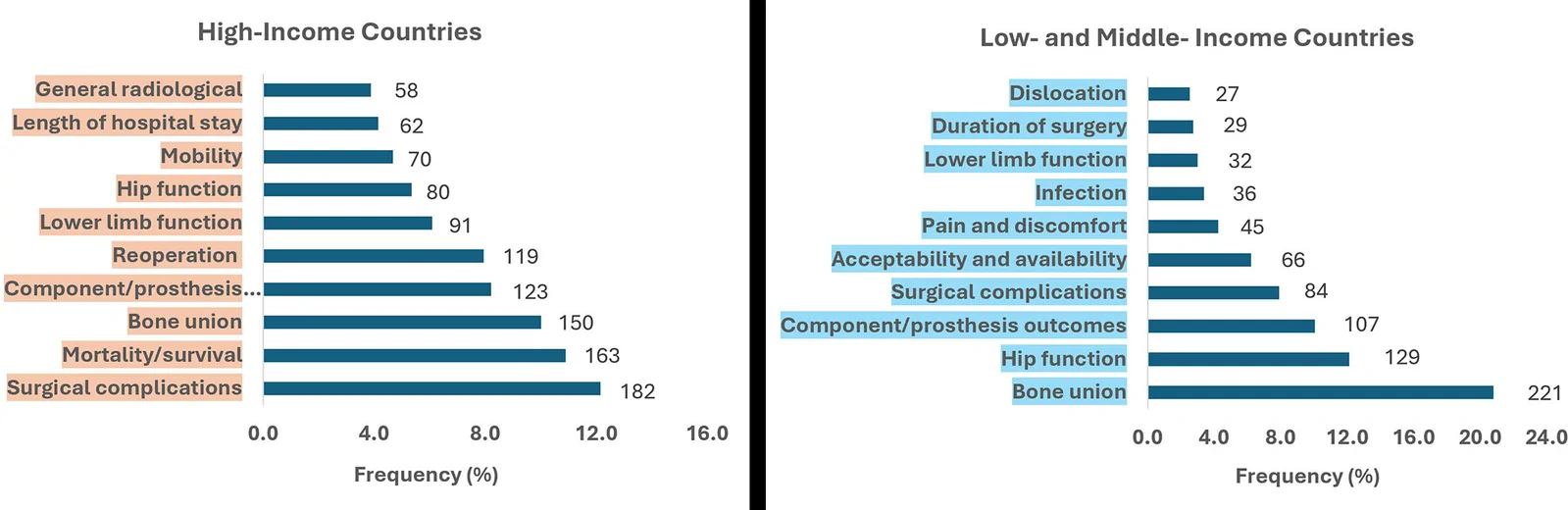
Frequently reported outcomes for postoperative periprosthetic femoral fracture patients in high-income countries and low- and middle-income countries.

Examining reported follow-up times for mortality/survival outcomes, the most frequently reported follow-up time was one year (75; 38.7%), and the longest reported follow-up period was up to 20 years (Figure 5).

**Fig. 5:**
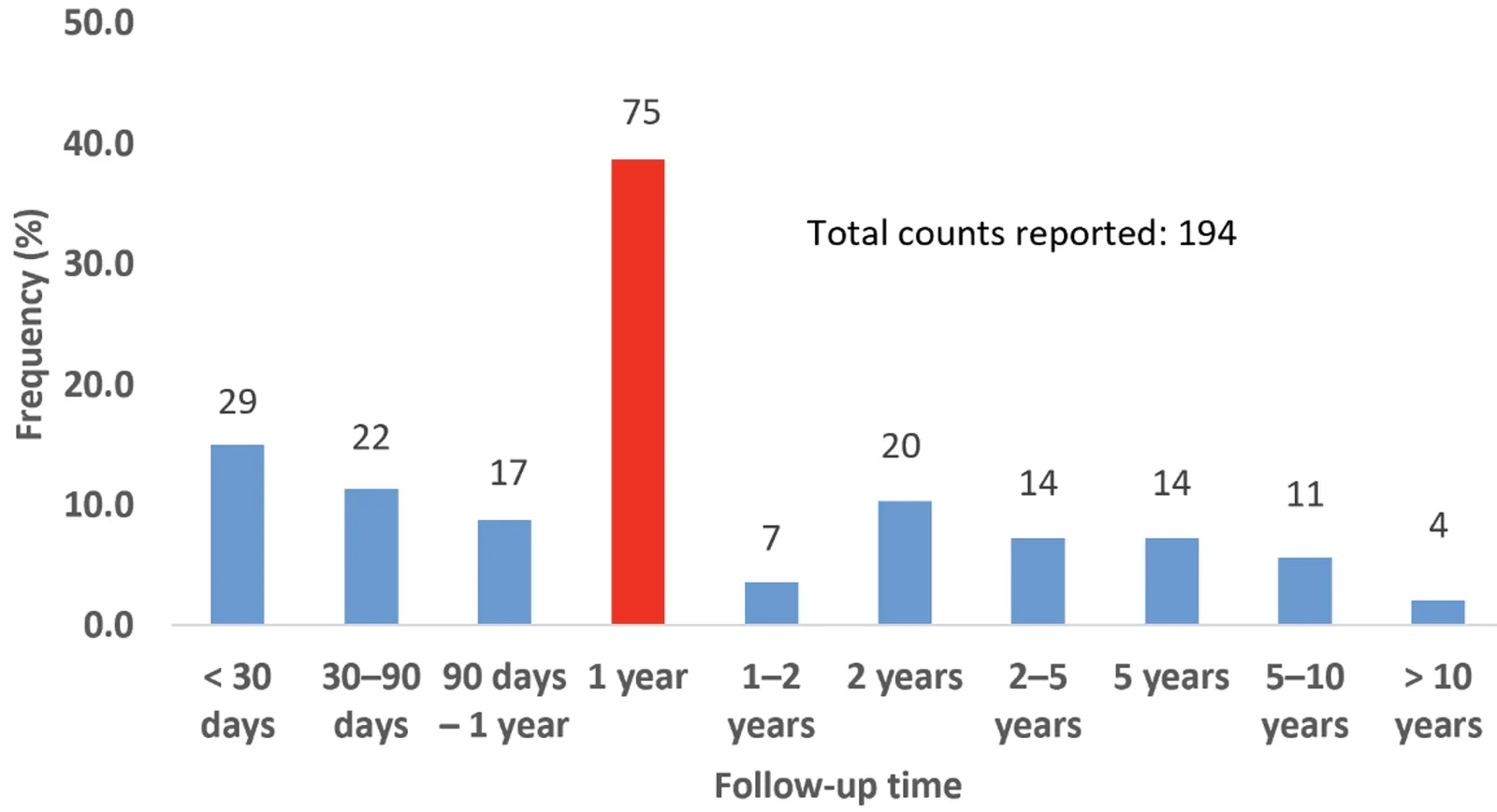
Frequently reported follow-up times for mortality/survival in postoperative periprosthetic femoral fracture patients.

### Outcome measurement instruments reported

In total, 858 individual OMIs were reported, comprising 387 outcome definitions and 471 patient- or clinician-reported outcome measures, including physical performance measures (Table III). Only 46.4% of outcomes reported were defined or detailed an OMI; where definitions were given, they were vague or lacked consistency. Of the 392 studies that reported measuring bone union, only 29 (7.4%) clearly defined how to measure it, 176 (44.9%) gave vague/non-measurable definitions, and 187 (47.7%) gave no definition or OMI (Supplementary Material 4). The Harris Hip Score^12^ and the Knee Society Score^13^ were the most frequently reported OMIs for periprosthetic hip and knee fracture, respectively, in studies from both HICs and LMICs (Table IV).

**Table IV.** Reported hip and knee outcome measurement instruments and their frequency across studies stratified by country income level: high-income countries (HICs) and low- and middle-income countries (LMICs).

Variable	HICs, no. studies (%)	LMICs, no. studies (%)	Total number of studies reporting OMI (%)
**Hip OMI**			
Harris Hip Score	57 (71.3)	126 (97.7)	183 (86.3)
Oxford Hip Score	10 (12.5)	N/A	10 (4.7)
Merle d’Aubigné and Postel Scoring System	3 (3.8)	N/A	3 (1.4)
Hip Disability and Osteoarthritis Outcome Score, Joint Replacement	4 (5.0)	N/A	4 (1.9)
Forgotten Joint Score (FJS-12)	3 (3.8)	N/A	3 (1.4)
Hip range of motion	N/A	1 (0.8)	1 (0.5)
Western Ontario and McMaster Universities Osteoarthritis Index	1 (1.3)	1 (0.8)	2 (0.9)
Gait analysis	1 (1.3)	N/A	1 (0.5)
Recorded according to Janda	1 (1.3)	N/A	1 (0.5)
University of California, Los Angeles Activity Score	1 (1.3)	N/A	1 (0.5)
Harris Pain Score	2 (2.5)	N/A	2 (0.9)
Hospital for Special Surgery Hip Score	N/A	1 (0.8)	1 (0.5)
Total studies	83	129	212
**Knee OMI**			
Knee Society Score	20 (62.5)	4 (25.0)	24 (51.1)
Oxford Knee Score	3 (9.4)	N/A	3 (6.4)
Knee Injury and Osteoarthritis Outcome Score	1 (3.1)	N/A	1 (2.1)
Hospital for Special Surgery Knee Score	N/A	6 (37.5)	6 (12.8)
Western Ontario and McMaster Universities Arthritis Index	2 (6.3)	N/A	2 (4.3)
Knee Society Functional Score	1 (3.1)	3 (18.8)	4 (8.5)
Knee Injury and Osteoarthritis Outcome Score for Joint Replacement	1 (3.1)	N/A	1 (2.1)
Kolmert Functional Criteria	1 (3.1)	N/A	1 (2.1)
Knee range of motion	1 (3.1)	N/A	1 (2.1)
Numerical mobility score (1 to 4)	1 (3.1)	N/A	1 (2.1)
Knee Society Knee Scoring System	N/A	1 (6.3)	1 (2.1)
Knee Society Pain Score	1 (3.1)	N/A	1 (2.1)
New York Hospital for Special Surgery knee score	N/A	1 (6.3)	1 (2.1)
Total studies	32	15	47

N/A, not available; OMI, outcome measurement instrument.

Across all included studies, health-related quality of life (HRQoL) was measured 39 times (in 6.9% of the included studies) using seven different instruments. The most frequently used instruments were the 12- and 36-Item Short-Form Health Survey questionnaires (SF-12 and SF-36), followed by the EuroQol five-dimension questionnaire (EQ-5D), which were reported 11 times each, respectively (Table V).

**Table V.** Health-related quality of life measurement instruments and their frequency of reporting across studies reporting outcomes on periprosthetic fractures of the hip, knee, and mixed.

Health-related quality of life measures	Hip, no. studies (%)	Knee, no. studies (%)	Mixed, no. studies (%)	Total, no. studies (%)
12-Item Short-Form Health Survey	7 (26.9)	3 (30)	1 (33.3)	11 (28.2)
36-Item Short-Form Health Survey	7 (26.9)	2 (20)	2 (66.7)	11 (28.2)
8-Item Short-Form Health Survey	1 (3.8)	N/A	N/A	1 (2.6)
EuroQol five-dimension questionnaire	8 (30.8)	3 (30)	N/A	11 (28.2)
Short Musculoskeletal Function Assessment – Dysfunction Index	N/A	2 (20)	N/A	2 (5.1)
Veterans Rand 12-Item Health Survey	2 (7.8)	N/A	N/A	2 (5.1)
Karnofsky Score	1 (3.8)	N/A	N/A	1 (2.6)
Total studies	26	10	3	39

N/A, not available.

## Discussion

Across 564 studies, including 119,445 patients, marked heterogeneity was found in the outcomes which were reported and how they were measured. Reporting of clinical, objective, surgeon-centred outcomes dominated, particularly bone- and implant-related outcomes, while patient-centred outcomes, measured using PROMs and HRQoL tools, were rarely reported. Less than half of all outcomes were accompanied by a clear definition or named OMI. The reporting of bone union epitomized this problem: despite being the single most frequently reported outcome domain subcategory (392 counts; 14.5% of all reported outcomes), only 7.4% of studies clearly defined how union was assessed; 44.9% provided vague, non-actionable definitions; and 47.7% provided no definition or OMI at all. HRQoL was measured in only 6.9% of included studies using seven instruments, most commonly the SF-12/36 and EQ-5D. The patterns in outcome reporting are consistent with a predominantly observational evidence base, relying on routinely collected data that were likely easy to access retrospectively, but which do not necessarily reflect what matters most to patients during recovery.

The outcomes identified in this review mirror those seen across similar orthopaedic lower-limb trauma studies. Outcomes reported in the open lower-limb fracture (OLLF) literature were also heterogeneous, dominated by objective clinical measures (e.g. bone union/infection), with infrequent use of PROMs or HRQoL tools.^14^ Similarly, the ankle/distal tibia trial literature demonstrates substantial inconsistency in outcome selection, assessment methods, and timing, with clinically focused domains reported far more often than longer-term, patient-centred outcomes.^15^

Taken together, the periprosthetic, OLLF, and ankle fracture literature converge on the same message: without standardized definitions and agreed OMIs, heterogeneity persists in terms of what and how we measure outcomes, leading to outcome reporting bias, obstructing robust evidence synthesis, and slowing progress towards better patient-centred care.^14,15^

The over-reliance on surgeon-important, objective clinical outcomes (e.g. bone union, component status, reoperation) likely reflects the retrospective, observational nature of the majority of included POPFF studies. Continuing the status quo risks a disconnect between the outcomes we report and those that are likely to be of value to patients, e.g. pain relief, function, participation, independence, and broader HRQoL. As in OLLF and ankle fracture, the infrequent and inconsistent use of PROMs/HRQoL measures in POPFF studies limits the ability to compare treatments using outcomes that best capture a successful recovery from the patient perspective.

A COS for POPFFs will address these issues by defining a minimum, stakeholder-agreed panel of outcomes and their preferred OMIs. In line with prior COS work in trauma, a POPFF COS should balance objective clinical endpoints (e.g. union, infection, reoperation, implant survival) with patient-reported outcomes (e.g. physical function and HRQoL) and provide clear definitions or recommend OMIs to support a unified approach to measuring core outcomes.

A key strength of this review is its breadth and inclusivity: 8,592 records were screened, and 564 full-text articles were included for data extraction, spanning multiple regions and care settings. Importantly, we incorporated Chinese-language databases. Given the scale and growth of research output from China, the inclusion of this literature enhances global relevance and mitigates language bias, a crucial step towards an internationally adoptable POPFF COS.

Study inclusion was restricted to a ten-year window, and an inevitable lag occurred between the literature search and publication. This was an intentional, pragmatic choice: ten years of POPFF literature yielded 564 studies for full-text review and data extraction – an ambitious undertaking that required time to deliver high-quality data extraction and quality control. Given the large study count and the authors’ experience in COS development projects, it is unlikely that extending the inclusion time window would have identified additional outcome domain subcategories of potential relevance for inclusion within a COS (as opposed to more synonyms). Notably, a previous review on outcomes reported in OLLF employed a staged search strategy and found that the search update did not generate any new outcome domain subcategories, empirically supporting the notion of outcome saturation over time.^14,15^

Study methodological quality was not appraised for inclusion to ensure capture of all potentially relevant outcome concepts, with the objective of cataloguing outcomes and OMIs rather than estimating effect sizes of interventions. In line with other reviews on outcome reporting, mapping and categorizing outcomes/OMIs to taxonomies or health frameworks involves subjective judgement. Recurring patterns (dominance of objective measures, sparse PROMs/HRQoL, variable definitions) found in this study are consistent with similar work on outcome reporting in orthopaedic trauma.^14,15^

This systematic review highlights substantial inconsistencies in outcome selection and measurement for POPFF, with a pervasive failure to capture PROMs and HRQoL and an over-reliance on surgeon-centred, objective clinical measures such as bone union. These findings mirror those in OLLF and ankle fracture, and together make a compelling case for a POPFF COS and a recommended set of unified OMIs. Standardizing what and how we measure will strengthen the evidence base required to optimize treatment strategies for this growing patient population, and to deliver outcomes that matter to patients. A POPFF COS should specify preferred OMIs with operational definitions to improve reproducibility and enable high-quality meta-analysis.

## Data Availability

All data extraction forms and any other materials used in the review are available on reasonable request.
